# Micro-mirror aided mid-infrared plasmonic beam combiner monolithically integrated with quantum cascade lasers and detectors

**DOI:** 10.1515/nanoph-2024-0688

**Published:** 2025-06-23

**Authors:** Georg Marschick, Mauro David, Xaver Gsodam, Nikola Opačak, Dominik Koukola, Elena Arigliani, Axel Evirgen, Virginie Trinité, Salvatore Pes, Stefania Isceri, Hermann Detz, Werner Schrenk, Aaron M. Andrews, Bernhard Lendl, Benedikt Schwarz, Gottfried Strasser, Borislav Hinkov

**Affiliations:** Institute of Solid State Electronics & Center for Micro- and Nanostructures, 27259 TU Wien, Wien, Austria; III-V Lab, A Joint Thales, Nokia and CEA-LETI laboratory, Palaiseau, France; CEITEC, Brno University of Technology, Brno, Czech Republic; Current Address: Department of Applied Mathematics & Physics, University of Applied Sciences Technikum Wien, Vienna, Austria; Institute of Chemical Technologies and Analytics, TU Wien, Wien, Austria; Silicon Austria Labs, Villach, Austria

**Keywords:** mid-infrared, photonic integrated circuit, monolithic integration, plasmonics, quantum cascade laser, quantum cascade detector

## Abstract

The development of novel mid-infrared (MIR) devices and systems is crucial for addressing applications in biomedical analysis, chemical reaction-monitoring, or high-bitrate free-space telecommunication. Combining multiple functional elements on one chip into complex miniaturized photonic integrated circuits (PICs), is the next step in these developments, yet limited by existing material and technology constraints. In this work, we introduce a new concept for realizing fully monolithic MIR-PICs based on low-loss on-chip plasmonic guiding and beam combining. The core of our study demonstrates a monolithic beam combiner by integration of active quantum cascade (QC) devices at ∼8 µm (laser and detector) with tailored passive waveguides based on weakly-coupled Ge/Au plasmonics and on-chip micro-mirror optics. The on-chip gold-coated micro-mirrors enhance the directional control and beam combining capabilities of the plasmon waveguides while minimizing energy dissipation typically associated with tight plasmon confinement. We discuss the MIR-PIC beam combiner design, micro-fabrication, and characterization and compare it to the routing concept of simple plasmonic Ge/Au y-couplers exploiting strong-confinement.

## 1 Introduction

Energy-efficient electronics (E3) is a long standing research field established with the first transistor [1] and followed by decades of development of micro- and nano-scale electronic devices. Their continuous increase in functional density and energy efficiency led to highly integrated central processing units (CPUs) with more than 130 billion individual transistors [2]. In contrast, energy-efficient photonics (E^2^P, also “Green Photonics”, exploiting till sub-wavelength structures [3]) is still in a much earlier stage of development and photonic integrated circuits (PICs), i.e. fully chip-scale photonic devices, have not yet reached a similar level of maturity and integrated functionality as their electronic counterparts. In addition, PIC development is currently still strongly focused on near-infrared (NIR) devices for telecom applications [4], [5], [6], [7], [8]. This leaves out the important mid-infrared (MIR) spectral range, which hosts many fundamental molecular absorptions [9], [10], [11], [12], [13], [14], [15], [16], [17] and simultaneously covers multiple atmospheric transparency windows suitable for telecommunication applications [18], [19], [20]. However, the bulky nature of the existing MIR systems poses several limitations including thermal challenges for mobile applications and prevents miniaturized setups as needed in *in-situ* spectroscopy [21], [22].

MIR-PICs offer a solution to these challenges. They are intrinsically more robust, cost-effective and show a drastically reduced power consumption (1-2 orders of magnitude) as compared to lab-scale MIR instrumentation such as the well-established Fourier Transform Infrared (FTIR) spectrometers. In addition, MIR-PICs unlock unprecedented capabilities based on combining chip-scale active QC components [23] like quantum cascade lasers (QCL) [24], [25], [26] and detectors (QCD) [27], [28] with passive components such as waveguides and modulators. They include beam-directing (with much better control than single-element QCLs [29]), shaping and modulating and thus hold immense potential for cost-efficient sensors and free-space telecom devices [30], [31]. Passive MIR waveguides are pivotal parts of such novel PIC architectures. Traditionally, dielectric waveguides based on total internal reflection and utilizing epitaxially-grown layer structures are employed, resulting in low-loss characteristics [32], [33], [34], [35]. But their typically strong mode confinement makes them less suitable for on-chip sensing and spectroscopic applications [36], [37], [38].

In contrast to that, recent advances in MIR plasmonic waveguides, such as dielectric-loaded or semiconductor-loaded surface plasmon polariton (DL/SLSPP) structures, offer a promising alternative [21], [39], [40], [41]. They can be tailored by thin (∼100s of nanometer) dielectric/semiconductor layer stripes to adjust the vertical mode extension for over 95 % of the mode being efficiently propagated in the surrounding medium [42]. The drawback of this configuration is the low modal index which results in very poor mode guiding capabilities along bent waveguide structures on the chip surface. In contrast, thick dielectric/semiconductor ridges (∼micrometer-scale) on top of the plasmonic metal layer significantly reduce the modal overlap with the surrounding medium, while simultaneously enabling effective routing capabilities, which is a crucial characteristic for realizing complex MIR-PIC geometries. However, finding suitable dielectric or semiconductor materials transparent up to wavelengths of *λ* = 12 µm that are also compatible with state-of-the-art MIR photonic processing technology is a challenging task [43], [44]. While it is true that plasmonic waveguides exhibit higher losses compared to dielectric waveguides, their flexibility in integrating both sensing and guiding sections, due to better mode profile matching, offers significant advantages for compact and multifunctional designs. Recently, we demonstrated the implementation of the polymeric material linear low-density Polyethylene (PE) on Au in the thick-ridge geometry, achieving the first proof of efficient MIR plasmonic mode-guiding along the chip surface [45]. However, the integration of PE polymer layers with QCL and QCD ridge structures is challenging due to their sensitivity to ambient conditions during fabrication which significantly complicates their integration into monolithic MIR-PICs.

In this work, we study two different integrated plasmonic designs for enabling MIR on-chip guiding and show their successful integration with QCLs and QCDs. We introduce a new MIR approach with plasmonic waveguiding to achieve versatile routing capabilities while preserving low-loss propagation by the integration of monolithic micro-mirror optics. The main advantages of this approach are given by its integration flexibility (very little dependence on the underlying substrate), compactness (micro- to millimeter scale waveguide dimensions with barely increased overall device footprint), fabrication simplicity (full compatibility with state-of-the-art MIR fabrication technology as e.g. for QCLs and QCDs and no need for complex steps such as epitaxial regrowth as in the competing dielectric waveguides [33]) and direct use in on-chip sensing experiments (through fully tailorable modal overlap with the surrounding medium). More details on the plasmonic waveguide concept for the MIR can be found in e.g. in: [41], [46].

As a proof-of-concept of a complex MIR-PIC, we demonstrate in this work a fully monolithic QC-based integrated MIR beam combiner. This device architecture represents an alternative approach to our previously demonstrated interband cascade (IC)-based device [47] and opens the path for longer wavelength applications of more advanced devices like heterodyne receivers.

## 2 Experimental section

### 2.1 Design and simulation

**Figure 1:**
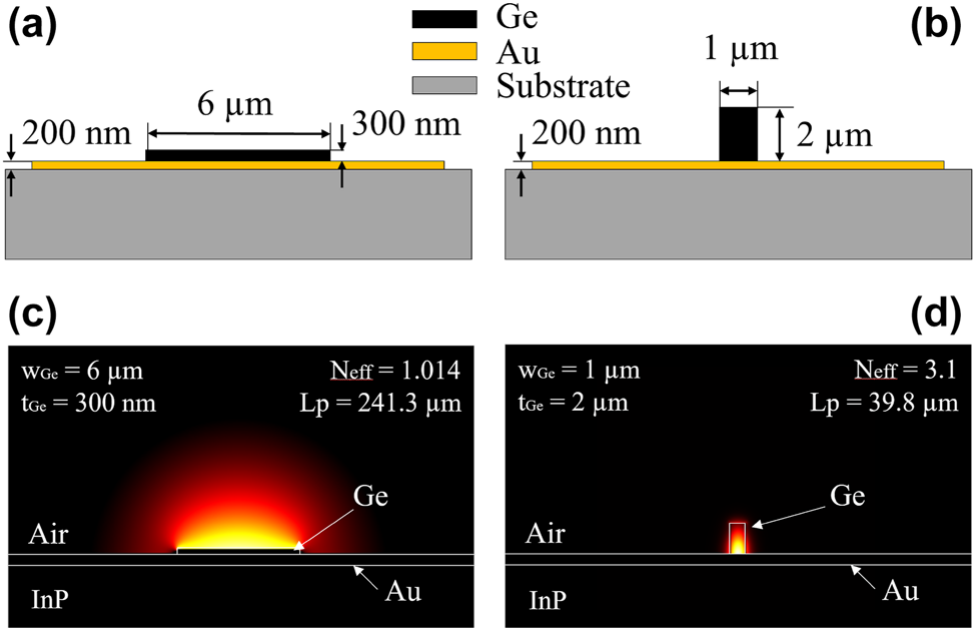
Cross sections of the proposed SLSPP waveguides: (a) schematic illustration and (c) simulation of a Ge-based plasmonic waveguide (thin configuration) with a weakly confined mode. (b) schematic illustration and (d) simulation of a Ge ridge SLSPP waveguide with a highly confined mode (thick configuration).

In order to evaluate the optical characteristics of SLSPP waveguides (WGs) based on Ge-layers on Au, we conducted finite element method (FEM) based simulations analyzing the resulting effective mode confinement. A careful study yields two distinct geometries, each offering unique advantages, illustrated in Figure 1. It shows the cross-section view sketches of both geometries in (a) and (b) together with the simulation results of the corresponding TM-polarized modes in (c) and (d): the first geometry (Figure 1(a)) provides weak vertical mode confinement, making it suitable for sensing applications due to its large modal overlap with the surrounding medium (Figure 1(c)) and low losses, while the second geometry offers sub-wavelength mode confinement (Figure 1(b) and (d)) and potentially good guiding/routing capabilities. The former is achieved by a thin and wide Ge waveguide layer, while the latter is best featured by a thick and narrow Ge waveguide ridge. TM is the polarization for direct light coupling from QC devices and, therefore, relevant in this case.

To carefully predict and optimize the geometrical factors to minimize the optical losses prior to device fabrication, the Wave Optics Module within the COMSOL Multiphysics was utilized.

**Figure 2:**
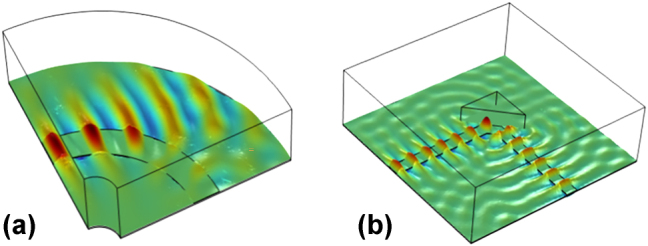
Simulation of the electric field component in z-direction for two distinct cases: Thin Ge-WG configuration with (a) a 90° curved waveguide and (b) two perpendicular waveguide sections (90° angle) employing an additional gold-covered micro-mirror.

To obtain precise predictions of the waveguide losses, we conducted simulations using material parameters extracted from ellipsometry and atomic force microscopy (AFM) measurements of fabricated layers using the same fabrication protocol as for the final integrated WGs. The higher losses presented here surpass those in previous studies using sputtered Au [40], [48] due to the increased roughness of the evaporated gold layer beneath the Ge stripe in this case [49]. In the configuration employing the thick Ge-WG with optimized dimensions of 1 × 2 µm (width x thickness), the SPP mode is effectively confined within the Ge-ridge due to the high material refractive index in the long-wave IR (LWIR) of approximately *n* = 3.8. The pronounced confinement and resulting large effective mode index of *N*_*eff*_ = 3.1 consequently facilitates efficient routing of the SPP modes along the chip-surface, potentially even around sharp bending angles [50]. However, this confinement comes at the cost of substantial interaction between the SPP mode and the bottom plasmonic Au layer, leading to remarkably high ohmic losses of 0.11 dB/μm even in a simple linear WG ridge (*λ* ∼ 8.2 µm). Despite the general possibility of adjusting this design to provide good mode guiding in non-straight geometries, the overall losses are very high, calling for the investigation of alternative solutions for guiding. In contrast, the geometry featuring the thin Ge-SLSPP WG (width x thickness: 6 µm × 300 nm) exhibits significantly lower losses, approximately 0.016 dB/mm for a straight WG. Additionally, the larger mode supported by the thin Ge/Au architecture, facilitates efficient coupling of the optical mode between the plasmonic and the laser/detector waveguides. The effective refractive index of the mode in this configuration is close to unity (*N*_*eff*_ = 1.014), suggesting weak confinement to the WG. Therefore, very poor mode guiding capabilities along WG-bends can be expected, which proves the initially mentioned impracticality of this geometry for complex MIR PICs. However, here we propose an innovative solution that enables on-chip mode-guiding while maintaining the advantages of the thin Ge SLSPP configuration. This approach reduces the losses and simplifies the fabrication compared to the challenges posed by the geometry with thick Ge. Building on the plasmonic reflection planes for 90° plasmon redirection demonstrated by Markey et al. [51], our approach introduces a novel strategy that integrates monolithic micro-optical structures with both active and passive components at the chip scale. As illustrated in the mode propagation simulation in Figure 2(a), a circular bent thin Ge SLSSP WG couples barely any light along its curvature. Instead, the introduction of an additional vertical gold-coated mirror structure, very efficiently couples the vast majority of the light even for an angle of 90° between the two arms of the plasmonic waveguide (see Figure 2(b)).

**Figure 3:**
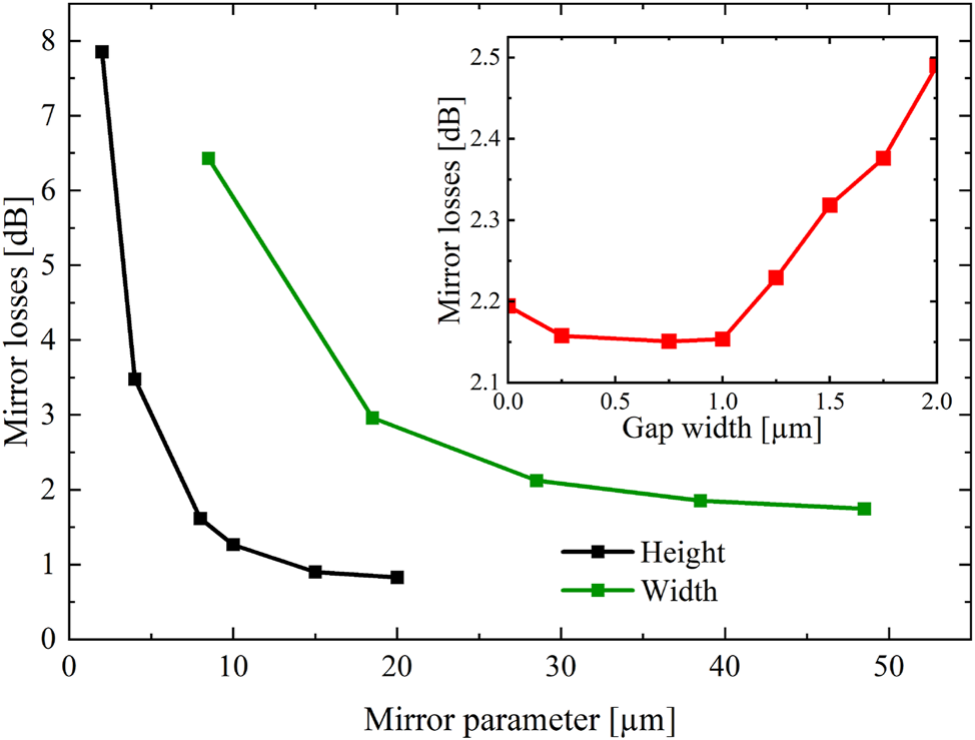
Simulated losses of a Au-coated micro-mirror with differing mirror parameters: SLSPP WG-mirror gap (red), mirror width (green), mirror height (black).

**Figure 4:**
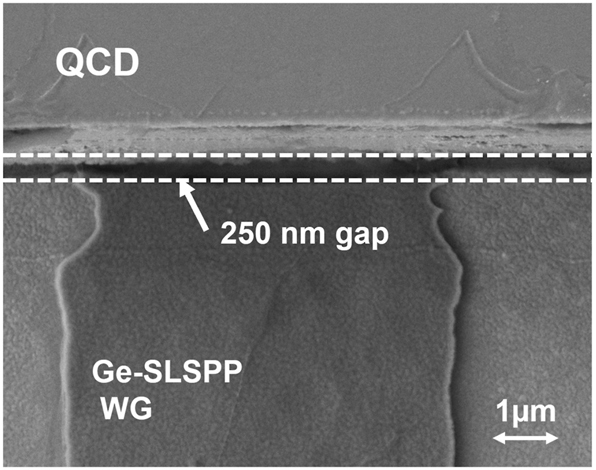
Fabricated gap between SLSPP-WG and on chip QCD through standard lithography and optical marker alignment with high precision. The gap-width here, highlighted through the two white dashed lines, is 250 nm.

The geometrical parameters of the mirror structures show a significant impact on the reflection losses in the simulations as shown in Figure 3. The achievable maximum mirror height of 8 µm, limited by the deep etch of the QCL/QCD structure itself, yields acceptable losses. For the lateral mirror dimension, simulations indicate low losses for values above 25 µm. The gap between the SPP-WG and the Au-coated mirror structure shows less significant impact on the mirror losses and gap widths below 1 µm, as seen in Figure 4 are easily feasible with appropriate lithography alignment. The simulation results in Figure 3 were achieved by varying one parameter while the others were kept constant at 0.5 µm gap-width, 8 µm mirror height and 28.5 µm mirror width. Considering these results, the expected losses due to a single Au-covered mirror with technologically feasible parameters are estimated to be 2.2 dB. Comparing the thin (300 nm) Ge WG geometry (0.016 dB/μm) with two additional integrated gold mirrors (4.4 dB) to the thick (2 µm) Ge WG design (0.11 dB/μm), the former becomes already more advantageous for relatively short total WG lengths exceeding 47 µm.

We want to mention here, that all components that are used in this study (e.g. QCL, QCD, plasmonic waveguides, micro-mirrors) have been analyzed individually first, before integrating them into more complex on-chip devices. As described in [40] this works well for enough statistical data, but also has its limitations.

### 2.2 Device fabrication

**Figure 5:**
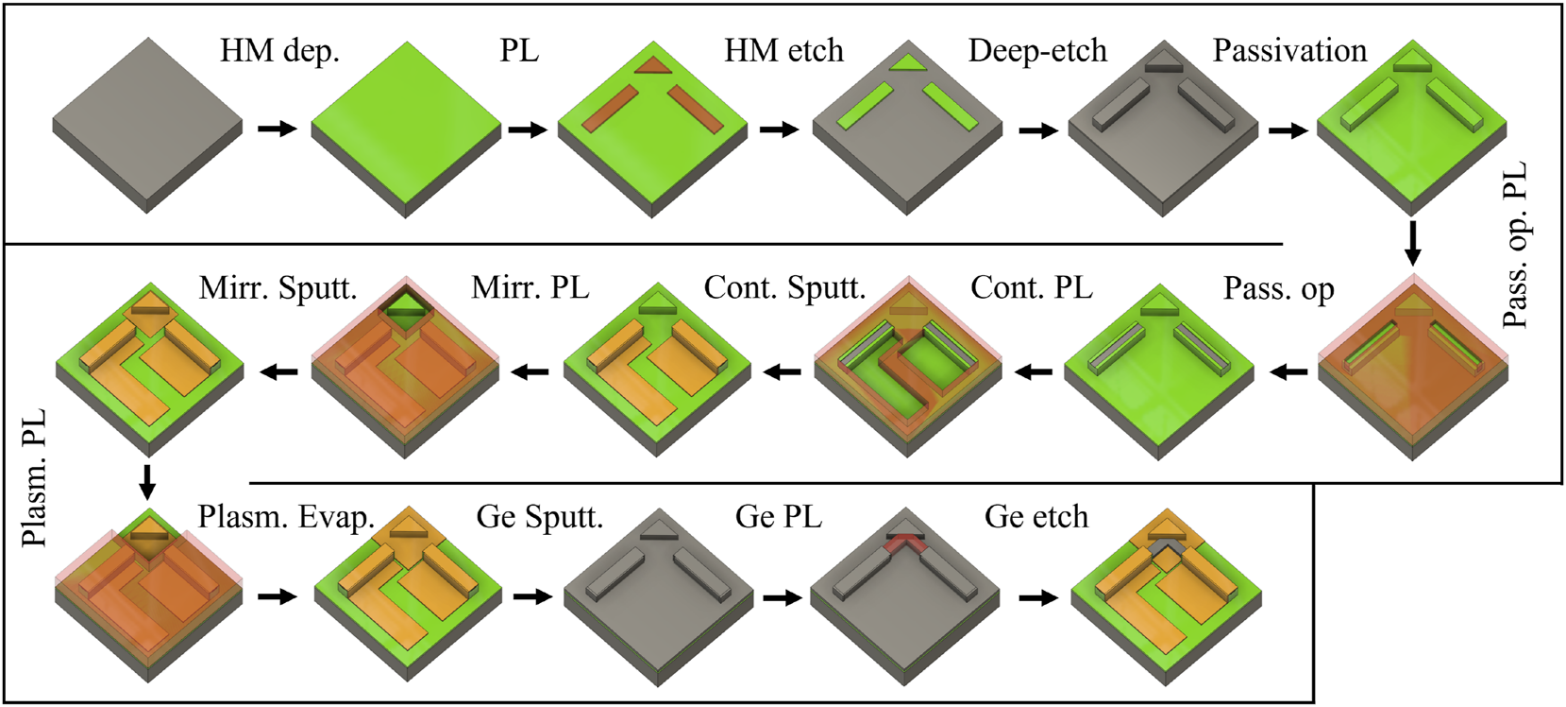
Process flow of the monolithic integrated laser-micro-mirror-detector structures at the example of a 90° configuration. The shown process steps are described in the main text. Abbreviations: hardmask (HM), photolithography (PL), passivation opening (Pass. op.), contact (Cont.), micro-mirror (Mirr.), sputtering (Sputt.), evaporation (Evap.), plasmonic-Au-layer (Plasm.).

**Figure 6:**
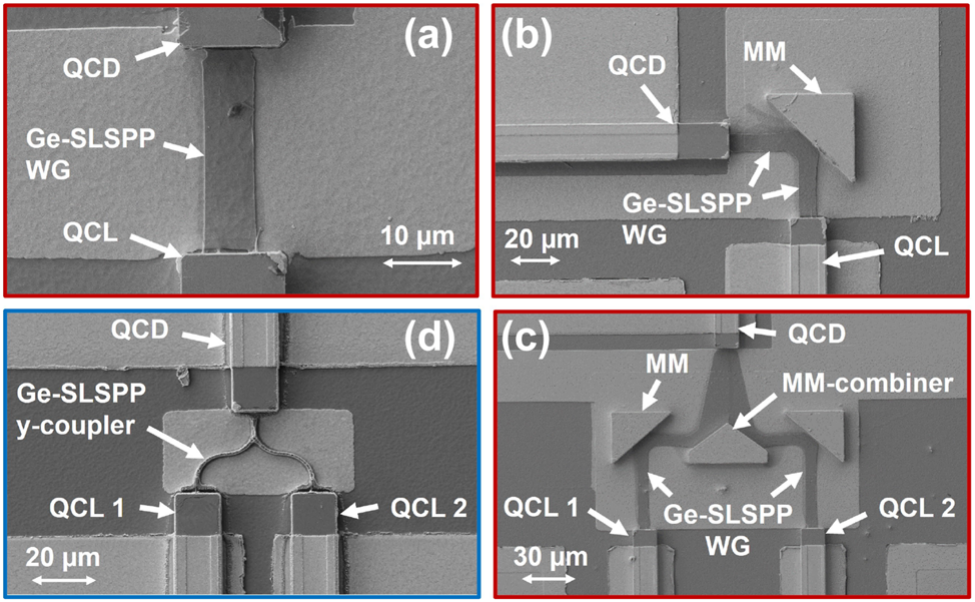
Scanning electron microscope (SEM) pictures of the different on-chip structures. The red-framed pictures show the thin Ge-WGs with micro-mirrors (MM), while the blued-framed one shows the thick Ge-WG with y-coupler geometry. Monolithic integrated: (a) QCL, QCD and thin straight Ge-SLSPP waveguide, (b) 90° arrangement of QCL and QCD with Ge-SLSPP waveguide and Au-coated micro-mirror, (c) spectral beam-combiner configuration consisting of two QCLs, a Ge-SLSPP waveguide guided by two 90° single mirrors and a center micro-mirror combiner with a combining angle of 100°, and a QCD, (d) spectral beam-combiner configuration consisting of two QCLs and a thick Ge-SLSPP y-coupler waveguide.

The realization and integration of those additional micro-mirror structures requires, besides establishing the process itself, careful alignment with the fabrication steps of the other on-chip components, such as the QCL and QCD. The fabrication procedure is schematically shown in Figure 5. It starts with the deposition of a 1 µm-thick layer of SiN, acting as a hardmask (HM), onto the epitaxial active region structure via plasma-enhanced chemical vapor deposition (PECVD). Subsequently, the SiN is patterned utilizing standard photolithography (PL), integrating structures for the corresponding QCL, QCD, and micro-mirrors. This step is finalized through CHF_3_ reactive-ion etching (RIE) of the SiN hardmask. Residual resist is removed using Acetone and Isopropanol, followed by a RIE deep etch in a Cl_2_–Ar chemistry to transfer the pattern from the SiN hardmask into the semiconductor material. Surface passivation and protection are achieved through a 250 nm-thick layer of SiN (PECVD). Top contact openings on top of the QCL/QCD ridges are created through another PL and RIE process. A subsequent PL step precedes sputtering of 10 nm of titanium (Ti) and 300 nm of Gold (Au) onto the sample for the top contacts, with undesired Au being removed in a lift-off process. To cover the sidewalls of the mirror structures with gold and without covering the laser/detector facets, a two-step PL-deposition process is necessary. First, we perform a PL for metallization of the mirror structures, ensuring that the laser and detector facets are protected with photoresist. The mirror structures are coated with 10 nm of Ti and 30 nm of Au via a sputtering process to ensure adequate sidewall coverage for efficient reflection of the plasmonic mode. This is followed by a second PL step to prepare for the subsequent anisotropic evaporation process of the plasmonic Au-layer (Ti: 10 nm/Au: 350 nm), which is optimized for metal deposition close to the facets without contaminating them, as an evaporation process more efficiently avoids coverage of vertical surfaces. After a lift-off process, 300 nm of Ge are sputtered, WGs are masked by PL, and WG definition is accomplished via oxygen-free SF_6_ etching in an inductively coupled plasma (ICP) RIE step. In the final step, the back facets of the QCLs are cleaved, and the devices are mounted on copper submounts and wire-bonded to printed circuit boards, rendering them ready for measurement. The processed structures are shown in Figure 6 including straight WGs in (a), 90° bends with a single mirror in (b) and quasi-s-bends with a center micro-mirror combiner in (c) with a combining angle of 100°. This configuration is the fundamental part of the spectral beam combiner. Figure 6(d) shows for comparison a y-coupler geometry where the thick Ge on Au configuration is used.

### 2.3 Optical characterization

In the previously presented simulations, defect-free device fabrication is assumed. Consequently, only optical losses arising from material properties and waveguide design are examined. However, in real devices, three sources contribute to optical losses associated with the waveguides: coupling losses (between the QCL/QCD and waveguide), waveguide losses due to attenuation of the electromagnetic field within the waveguide material, and scattering losses resulting from defects and imperfections during the fabrication process. To characterize the losses of the Ge-SLSPP waveguides, we employ a methodology similar to the effective cutback technique commonly utilized for waveguide characterization [52]. Instead of cleaving the chip, we fabricated waveguides of varying lengths on the same chip and measured each one individually. This method relies on the assumption that the optical properties are consistent across all waveguides, allowing us to obtain results comparable to the standard cutback technique. We used a calibration sample without a waveguide to normalize the measurements. Further details can be found in the Supplementary material. The used on-chip QCL was operated in pulsed mode (1 kHz, 1 µs pulse width) using an HP 8114A pulse generator. Signals from both the on-chip QCD and an external MCT aligned to the device back-facet were recorded simultaneously via a Tektronix TDS 2024C oscilloscope. Probing needles connected the pulse generator and oscilloscope to the top and bottom contacts of the QCL and QCD for measurements. The sample stage, including the copper sample holder, was maintained at 17 °C using an integrated Peltier cooler to ensure consistent conditions during testing. Various waveguide lengths were tested as part of the configurations.

## 3 Results and discussion

### 3.1 Optical characterization

**Figure 7:**
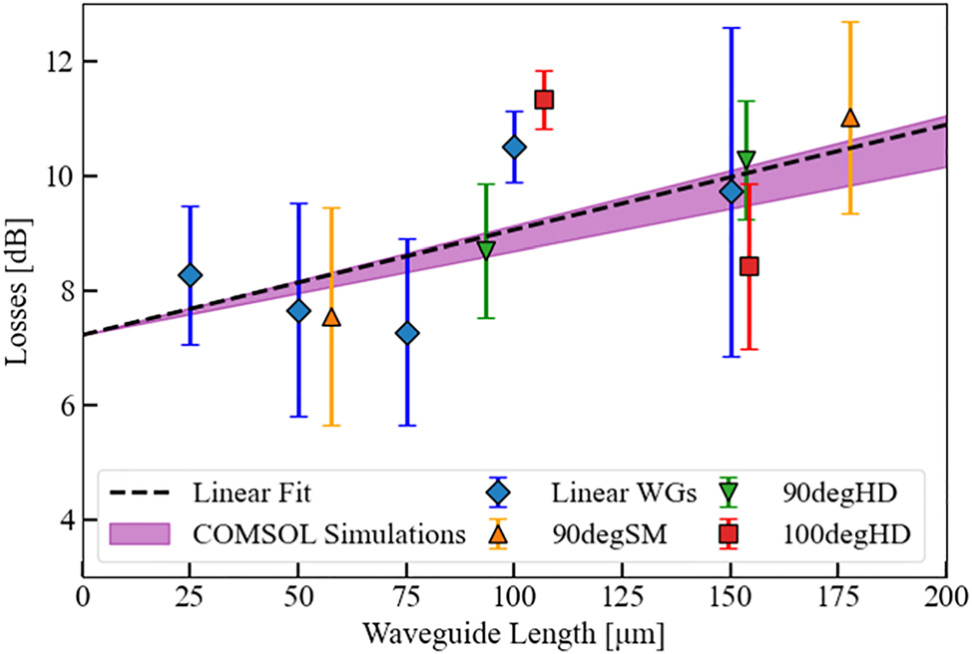
Experimental results showing $\alpha_{\mathrm{tot}}-\bar{\alpha_{M}}$ for different WG configurations (Linear WGs: linear waveguide configuration; 90degSM: 90°-angled single micro-mirror; 90/100degHD: 90°-/100°-angled double micro-mirrors, i.e. beam-combiner configuration). The color-coded data points correspond to the different designs depicted in Figure 6(a–c). Each data point encompasses at least three measurements from separate samples. The black dotted line is a linear fit of the linear WG configuration (blue datapoints) showing the waveguide losses *α*_*wg*_. The purple cone illustrates the simulation region resulting from a wavelength range variation between *λ* = 8–8.3 µm of the individual on-chip QCLs that are used for the measurements on every single chip. The crossing point of the linear fit with the *y*-axis for zero WG length corresponds to the SLSPP WG coupling losses (*α*_*c*_).

The summary of all measured losses for the different configurations from Figure 6(a–c) is presented in Figure 7 with the main values extracted in Table 1. Consistent with our simulation data shown in Figure 1(d), the losses of the thick Ge configuration are very high, even preventing proper experimental analysis of these devices. We attribute the inability to measure even the short (< 50 µm) thick Ge/Au plasmonic waveguide samples to the significant additional fabrication and coupling losses obtained as a consequence of the geometry. Therefore, we focus the further analysis on the thin-Ge plus micro-mirror samples, which not only show promising performance, but also enable effective measurements across various configurations.

**Table 1:** Comparison of the simulated (left column) and measured losses (right column) for the different analyzed geometries, i.e. micro-mirror, double micro-mirror (100° and 90°) and the waveguide (^a^Double mirrors were not simulated but assumed to show twice the mirror losses of a single mirror).

Losses	Simulation	Measurement
Micro-mirror	2.2 dB	7.9 dB
Double micro-mirror (100°)	4.4 dB^a^	14.8 dB
Double micro-mirror (90°)	4.4 dB^a^	20.1 dB
Coupling	–	7.2 dB
Waveguide	0.016 dB/μm	0.018 dB/μm

The blue diamonds in Figure 7 represent the mean values for the linear waveguide devices at various lengths (see Figure 6(a)), serving as the reference without mode redirection. A linear fit (black dotted line) reveals waveguide losses of *α*_*wg*_ = 0.018 dB/μm, while coupling losses (*α*_*c*_ = 7.2 dB) are extracted from the line’s zero offset. As the QCL devices were not wavelength-locked, slight wavelength variations (8–8.3 µm) were considered in the simulations, reflected by the widening purple cone. Colored data points show results for different integrated gold micro-mirror designs. To quantify mirror losses, total losses (*α*_*tot*_) were adjusted by subtracting coupling and waveguide losses. The discrepancy observed between the simulated (0.016 dB/μm) and actual (0.018 dB/μm) WG losses can again be reasonably attributed to imperfections in the waveguides and the underlying plasmonic Au layer. Mirror losses (*α*_*M*_) were then calculated, with experimental results showing approximately 5 dB higher losses than simulations for the single-mirror design. This discrepancy is likely due to fabrication imperfections, such as surface roughness, edge diffraction, and coating imperfections, all of which can increase scattering, absorption, and reduce reflectivity. Additionally, although SEM images suggest that the reflective surfaces are mostly vertical, slight deviations from perfect verticality could also contribute to the increased losses.

After demonstrating the effectiveness of the micro-mirror design, a critical component is the single s-bend or double s-bend, i.e. y-coupler. The later structure enables the combination of optical signals from two inputs into a single output (or vice versa), making it a fundamental building block for efficient signal routing. To optimize signal coupling, we designed and realized two combiner configurations: one with a 90-degree combining angle and the second one with a 100-degree angle and a tapered structure. A comparison of the simulated (left column) and measured losses (right column) for the various geometries – including the micro-mirror, double micro-mirror (100° and 90°), and the waveguide can be found in Table 1. The measured losses show that the double micro-mirror structure with a 100° combining angle (Figure 6(c)) exhibits twice the losses of a single mirror, while the 90° configuration shows nearly three times the losses. This is because the slightly larger angle allows for a smoother light transition between waveguides, reducing scattering and improving signal combination efficiency. Therefore the 100-degree design is more effective than the 90-degree one. Overall, these results demonstrate that adjusting the mirror angle can significantly improve the efficiency of the combiner. This proves that, through careful design, on-chip routing can be optimized to suit specific applications or requirements, offering flexibility in how light is managed and routed within the photonic circuit.

### 3.2 Monolithic mid-infrared beam combiner

**Figure 8:**
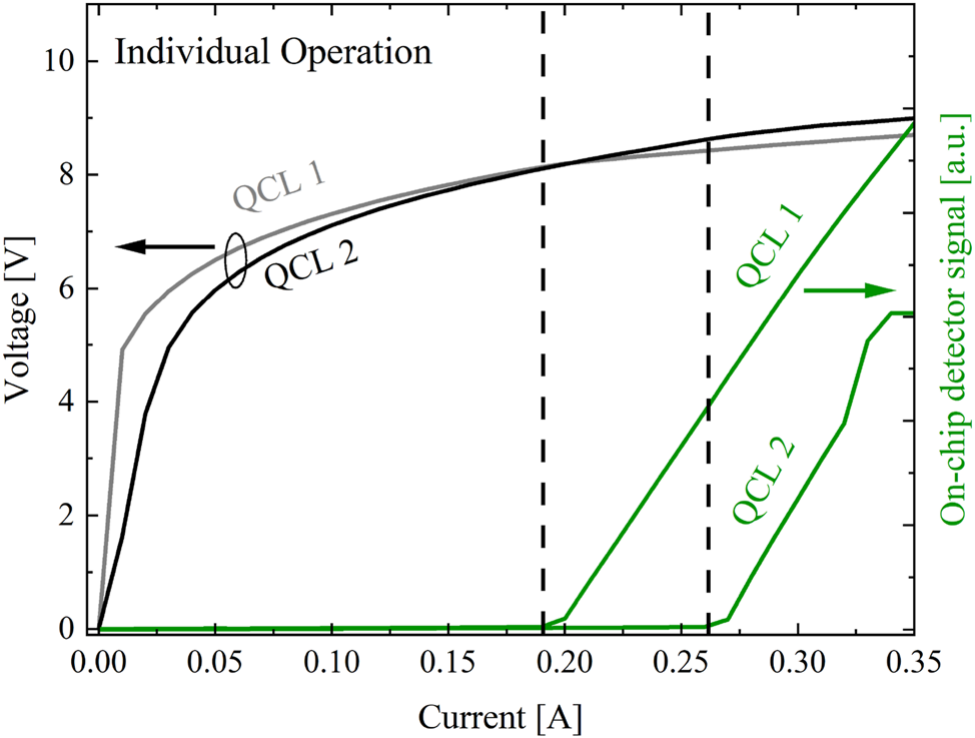
Individual CW light-current-voltage (LIV) characterization of QCL 1 and QCL 2 in the 90° beam combiner configuration (similar to Figure 6(c), but center mirror with 90° combining angle) measured with the on-chip QCD (green traces). The threshold currents are emphasized by the dotted lines.

**Figure 9:**
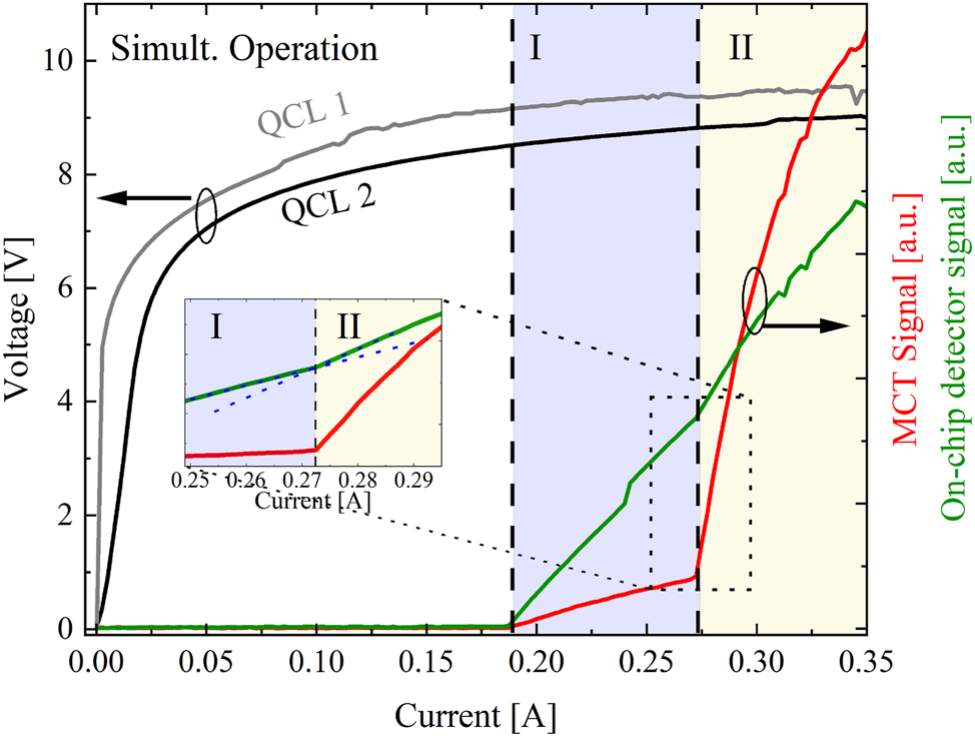
CW-LIV characterization of the monolithic beam combiner, showing the measured optical signals on the on-chip QCD (green trace) and the external MCT detector (red trace) for both QCLs being driven in parallel.

In this section, we present a proof-of-concept demonstration of a monolithic mid-infrared beam combiner utilizing micro-mirror-aided plasmonic guiding. The goal of these measurements is to validate the ability of our integrated design to combine light from two distinct lasers into a single on-chip detector. The significance of our findings is illustrated in Figures 8 and 9, which present an operational analysis of a dedicated sample using our beam combiner configuration with a 90° center mirror (design shown in Figure 6(c)). Here, the 90° center mirror structure was selected for the superior laser performance. The sample was cooled to a temperature of *T* = −194 °C within a cryostat to facilitate continuous-wave (CW) operation of the QCLs. Figure 8 shows light-current-voltage (LIV) data of both QCLs in individual operation detected via the on-chip QCD. Figure 9 shows the concurrent operation of both QCLs and confirms the simultaneous detection of both optical signals through the on-chip QCD. The lasing behavior can be categorized into two distinct regimes: Regime I (blue area) represents lasing of QCL 1 only. Due to the SiN/Au coating on the backside facet of QCL 1, the weak signal detected on the external MCT likely originates from emission through the substrate. In Regime II (yellow area), QCL 1 and QCL 2 are in the lasing regime simultaneously. Here, the back-facet of QCL 2 is uncovered, allowing its laser emission to be more efficiently out-coupled and detected with the MCT. A change in the slope of the on-chip detector photocurrent at QCL 2’s threshold current highlights this transition, as shown in the inset graph. The threshold current of QCL 2 is slightly higher during simultaneous lasing, likely due to thermal effects induced by QCL 1’s operation. These experiments demonstrate the significant role of the on-chip beam combiner in advancing MIR-PIC technology and show its potential in enabling the development of more complex PIC systems. For example, the integration of our beam combiner could facilitate sophisticated functionalities such as multi-channel signal processing and enhanced sensor applications, ultimately paving the way for innovative solutions in telecommunications, environmental monitoring, and biomedical diagnostics. This work lays a solid foundation for future research and development, demonstrating the impact of our on-chip beam combiners design in realizing the next generation of MIR photonic system.

## 4 Conclusions and outlook

In this work, we present the first integration of Au-covered monolithic micro-mirrors, thin Ge-based plasmonic waveguides, and QCL/QCD structures on a single epilayer chip. Through a combination of numerical simulations and experimental validation, we demonstrate the feasibility of using thin Ge/Au-based SPP waveguides integrated with Au-covered micro-mirrors for on-chip signal routing. While our approach does not surpass dielectric waveguides in performance, it demonstrates the feasibility of effectively achieving on-chip signal routing in monolithic MIR PICs. This integration lays the foundation for more advanced applications, such as high-speed heterodyne detection and on-chip Mach–Zehnder interferometry, with potential for miniaturized transceivers, and sensing systems in healthcare and environmental monitoring [50], [53], [54].

## Supplementary Material

Supplementary Material Details
