# Supplementary material for: Micro-mirror aided mid-infrared plasmonic beam combiner monolithically integrated with quantum cascade lasers and detectors
Source: Nanophotonics. 2025 Jun 23;14(21):3397–405. doi: 10.1515/nanoph-2024-0688 (PMC12552871; doi:10.1515/nanoph-2024-0688)
Supplement: Supplementary file 1 — Supplementary Material Details [file j_nanoph-2024-0688_suppl_001.pdf]

Supplementary Material

Georg Marschick, Mauro David, Xaver Gsodam, Nikola Opačak, Dominik Koukola, Elena Arigliani, Axel Evirgen, Virginie Trinité, Salvatore Pes, Stefania Isceri, Hermann Detz, Werner Schrenk, Aaron M. Andrews, Bernhard Lendl, Benedikt Schwarz, Gottfried Strasser, and Borislav Hinkov*

Micro-mirror aided mid-infrared plasmonic beam combiner monolithically integrated with quantum cascade lasers and detectors

1 Supplementary Material

1.1 Cutback Technique and Measurement Approach

To characterize optical losses, we used a method similar to the standard cutback technique. In traditional cutback, waveguides (WGs) are progressively shortened and measured at each length, allowing WG and coupling losses to be derived from a linear fit of the total measured losses as a function of inverse device length. In this study, we fabricated multiple WGs of varying lengths on the same chip and characterized them individually. This method, assuming consistent optical properties across WGs, yields comparable results to the standard cutback technique [1]. A calibration sample without a Ge-WG was fabricated to normalize the measurements. The sample, featuring a directly butt-coupled QCD and external QCL, had an air gap of approximately 2 µm. The losses attributed to the micro-mirrors (α_M) were calculated by subtracting the coupling losses ($\alpha_c = 7.2\,\mathrm{dB}$) and the waveguide losses ($\alpha_{wg} = 0.018\,\mathrm{dB/\mu m}$) from the total measured losses (α_{tot}) of the structures employing mirrors. Figure 7 in the manuscript shows the mean values of linear WG devices for various lengths, with blue diamonds representing the reference configuration without additional mode redirection. A linear fit to the data (black dotted line) reveals waveguide losses (α_{wg}) of $0.018\,\mathrm{dB/\mu m}$, while coupling losses (α_c) were determined to be 7.2 dB from the zero offset of the line. Variations in emitted wavelengths from the QCL devices (between 8 and 8.3 µm) due to the absence of wavelength locking were accounted for in the simulations, as seen by the widening purple cone in the plot. Colored data points correspond to designs incorporating integrated gold micro-mirrors for mode redirection. To quantitatively assess and compare losses from different micro-mirror designs, the total measured losses (α_{tot}) were adjusted by subtracting coupling and waveguide losses. The resulting mirror losses were calculated as follows:

$$\overline{\alpha_M} = \frac{1}{n}\sum_{i=1}^{n} \alpha_{tot_n} - \alpha_{wg} - \alpha_c$$

where n is the number of measured devices. Figure 7 in the manuscript illustrates $\alpha_{tot} - \overline{\alpha_M}$ alongside the standard deviation of α_{tot} at corresponding waveguide lengths. Different waveguide lengths for single and double mirror configurations stem from various design versions. Experimental results show that the mirror losses for the single-mirror configuration are about 5 dB higher than in simulations, likely due to fabrication imperfections such as non-fully vertical reflection planes of the gold layer, leading to increased reflection losses.

Georg Marschick, Mauro David, These Authors contributed equally to this work.
Georg Marschick, Mauro David, Xaver Gsodam, Nikola Opačak, Dominik Koukola, Elena Arigliani, Stefania Isceri, Werner Schrenk, Aaron M. Andrews, Benedikt Schwarz, Gottfried Strasser, TU Wien, Institute of Solid State Electronics & Center for Micro- and Nanostructures, Wien, Austria
Axel Evirgen, Virginie Trinité, Salvatore Pes, III-V Lab, a joint Thales, Nokia and CEA-LETI laboratory, Palaiseau, France
Bernhard Lendl, TU Wien, Institute of Chemical Technologies and Analytics, Wien, Austria
Hermann Detz, CEITEC, Brno University of Technology, Brno, Czech Republic; current address: University of Applied Sciences Technikum Wien, Department of Applied Mathematics & Physics, Vienna, Austria
***Corresponding author: Borislav Hinkov,** TU Wien, Institute of Solid State Electronics & Center for Micro- and Nanostructures, Wien, Austria & Silicon Austria Labs, Villach, Austria, borislav.hinkov@silicon-austria.com

References

[1] M. David, A. Dabrowska, M. Sistani, I. Doganlar, E. Hinkelmann, H. Detz, W. Weber, B. Lendl, G. Strasser, B. Hinkov, *Optics Express* **2021**, *29*, 26 43567.
